# Supplementary material for: Observation of cavity-tunable topological phases of polaritons
Source: Nat Commun. 2025 Jul 1;16:5914. doi: 10.1038/s41467-025-61121-5 (PMC12215189; doi:10.1038/s41467-025-61121-5)
Supplement: Supplementary file 1 — Supplementary Information [file 41467_2025_61121_MOESM1_ESM.pdf]

Supplementary Information for
Observation of cavity-tunable topological phases of polaritons

Dong Zhao,[1,*] Ziyao Wang,[1,*] Linyun Yang,[1] Yuxin Zhong,[1] Xiang Xi,[2] Zhenxiao Zhu,[1] Xiaoyuan Jiao,[1] Qing-an Tu,[1] Yan Meng,[2,†] Bei Yan,[3,†] Ce Shang,[4,†] and Zhen Gao[1,5,6,†]

[1] *Department of Electronic and Electrical Engineering, Southern University of Science and Technology; Shenzhen 518055, China.*
[2] *School of Electrical Engineering and Intelligentization, Dongguan University of Technology, Dongguan, 523808, China.*
[3] *College of Science, Wuhan University of Science and Technology, Wuhan 430081, China.*
[4] *Aerospace Information Research Institute, Chinese Academy of Sciences, Beijing 100094, China.*
[5] *State Key Laboratory of Optical Fiber and Cable Manufacture Technology, Southern University of Science and Technology; Shenzhen 518055, China.*
[6] *Guangdong Key Laboratory of Integrated Optoelectronics Intellisense, Southern University of Science and Technology; Shenzhen 518055, China.*

Contents

Supplementary Note 1. Theoretical Model 2
1.1 The dipolar Hamiltonian 2
1.2 The photonic Hamiltonian 3
1.3 The light-matter interaction Hamiltonian 4
1.4 The polaritonic Hamiltonian 6
1.5 Dipole-photon energy detuning 7
Supplementary Note 2. Phase transitions 7
2.1 SU(3) polaritonic model 7
2.2 SU(2) polaritonic model 7
Supplementary Note 3. Simulation and Experimental details 8
3.1 The microwave helical resonator mode 8
3.2 The cavity mode 8
3.3 The experimental scheme 9
3.4 The simulated and measured amplitude distributions of the right topological edge states 10
Supplementary Note References 10

This document includes:
Supplementary Sections 1 to 3
Supplementary Fig. 1 to 7

* These authors contributed equally
† Corresponding authors. Email: mengyan@dgut.edu.cn(Y.M.); yanbei@wust.edu.cn(B.Y.); shangce@aircas.ac.cn(S.C.); gaoz@sustech.edu.cn.(Z.G.)

Supplementary Note 1. Theoretical Model

For completeness, we follow closely the presentation of Ref. [1], providing details of the model. This model elucidates the intriguingphenomenon of polariton formation, as represented by Eq. (2) in the main text. Here, we provide a detailed account of the model, which characterizes a general configuration of dipoles. In this setup, each oscillating dp is defined by three fundamental parameters: the effective mass M, the effective charge Q of the dipole moment, and the resonance frequency ω_0. We consider these dps as a dimerized chain that is embedded within a cavity, as illustrated in Supplementary Fig. 1. The Coulomb interactions among these dipoles give rise to the emergence of collective dipolar excitations, and these excitations, in turn, engage in interactions with the photonic modes in the cavity and lead to the formation of polaritons. We formulate the model of polaritons as the Hamiltonian

$$H_{\rm pol} = H_{\rm dp} + H_{\rm ph} + H_{\rm dp-ph}, \tag{S1}$$

where $H_{\rm dp}$ is the dipolar Hamiltonian (Sec.1. 1.1), $H_{\rm ph}$ is the photonic Hamiltonian (Sec.1. 1.2), and $H_{\rm dp-ph}$ is the light-matter coupling Hamiltonian (Sec.1. 1.3).

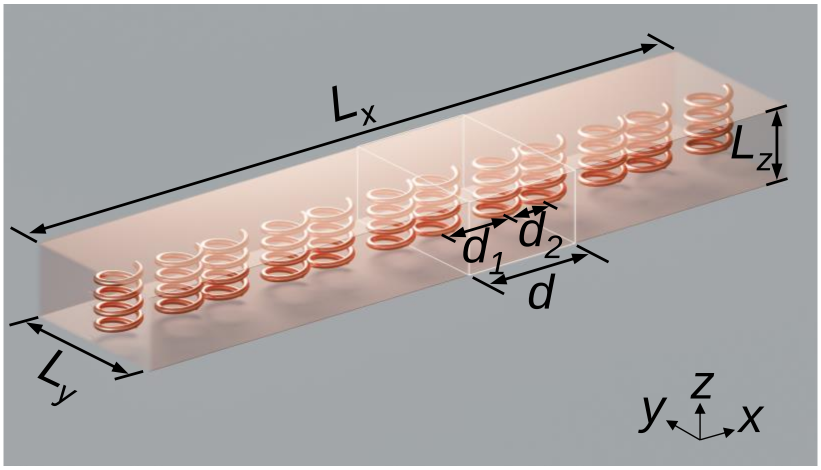

Supplementary Fig. 1 Sketch of the system. The chain consists of N dimers of dipoles, where the unit cell has two dipoles (Corresponding to sublattices A and B) associated with the resonance frequency ω_0 and the length scale a. The dimerized chain exhibits a period d, defined as the sum of d_1 and d_2.

1.1 The dipolar Hamiltonian

The dipolar Hamiltonian in Eq. (S1) is given by

$$H_{\rm dp} = H_{\rm dp}^0 + H_{\rm dp}^{\rm int}, \tag{S2}$$

where the noninteracting part ($\hbar = 1$ throughout the paper) reads as

$$H_{\rm dp}^0 = \sum_{n=1}^{N} \omega_0 \left(a_n^\dagger a_n + b_n^\dagger b_n \right), \tag{S3}$$

corresponding to the Hamiltonian for uncoupled harmonic excitations with resonance frequency ω_0. The index $n \in [1, N]$ denotes the number of dimers in the chain. In Eq. (S3), the bosonic operators a_n $\left(a_n^\dagger\right)$ and b_n $\left(b_n^\dagger\right)$ annihilate (create) an excitation with transverse polarization (oscillating in the z direction) in n-th dimer, where the dipole belongs to the A and B sublattices, respectively. This scheme is depicted in Supplementary Fig. 1. The long-range dipolar interaction between the excitations is governed by the term $H_{\rm dp}^{\rm int}$, which can be split into the intrasublattice Hamiltonian denotes the coupling between the same sublattices (A, A and B, B) and the intresublattice Hamiltonian denotes the coupling between the two inequivalent sublattices (A, B and B, A):

$$H_{\rm dp}^{\rm int} = H_{\rm dp}^{A,A\&B,B} + H_{\rm dp}^{A,B\&B,A}. \tag{S4}$$

The intrasublattice Hamiltonian reads as

$$H_{\mathrm{dp}}^{A,A\&B,B}=\sum_{n=1}^{N}\sum_{m=n+1}^{N}\frac{\Omega}{(m-n)^3}\left[\left(a_n^\dagger+a_n\right)\left(a_m^\dagger+a_m\right)+\left(b_n^\dagger+b_n\right)\left(b_m^\dagger+b_m\right)\right], \tag{S5}$$

and the intresublattice Hamiltonian reads as

$$H_{\mathrm{dp}}^{A,B\&B,A}=\sum_{n=1}^{N}\left[\sum_{m=n}^{N}\frac{\Omega}{\left(m-n+d_1/d\right)^3}\left(a_n^\dagger+a_n\right)\left(b_m^\dagger+b_m\right)+\sum_{m=n+1}^{N}\frac{\Omega}{\left(m-n-1+d_2/d\right)^3}\left(b_n^\dagger+b_n\right)\left(a_m^\dagger+a_m\right)\right]. \tag{S6}$$

Here we introduce the coupling constant $\Omega=\frac{{k_x}^2}{2M\omega_0 d^3}\ll\omega_0$ with the lattice constant $d=d_1+d_2$, and where d_1 and d_2 are the two central sites separations, as shown in Supplementary Fig. 1.
Employing the Fourier transforms $a_n=\frac{1}{\sqrt{N}}\sum_{k_x}\mathrm{e}^{\mathrm{i}nk_xd}a_{k_x}$ and $b_n=\frac{1}{\sqrt{N}}\sum_{k_x}\mathrm{e}^{\mathrm{i}nk_xd}b_{k_x}$ with the wavevector $k_x=2\pi p/Nd$ and the integer $p\in[-N/2,+N/2]$ (N should be even), the Eq. (S2) becomes

$$\begin{aligned}H_{\mathrm{dp}}=\sum_{k_x}\Big\{&\left(\omega_0+\Omega f_{k_x}\right)\left(a_{k_x}^\dagger a_{k_x}+b_{k_x}^\dagger b_{k_x}\right)+\frac{\Omega}{2}f_{k_x}\left(a_{k_x}^\dagger a_{-k_x}^\dagger+a_{-k_x}a_{k_x}+b_{k_x}^\dagger b_{-k_x}^\dagger+b_{-k_x}b_{k_x}\right)\\&+\Omega\left[g_{k_x}a_{k_x}^\dagger\left(b_{k_x}+b_{-k_x}^\dagger\right)+g_{k_x}^*\left(b_{k_x}^\dagger+b_{-k_x}\right)a_{k_x}\right]\Big\},\end{aligned} \tag{S7}$$

with

$$f_{k_x}=2\sum_{n=1}^{\infty}\frac{\cos(nk_xd)}{n^3}\quad\text{and}\quad g_{k_x}=\sum_{n=0}^{\infty}\left[\frac{\mathrm{e}^{\mathrm{i}nk_xd}}{\left(n+d_1/d\right)^3}+\frac{\mathrm{e}^{-\mathrm{i}(n+1)k_xd}}{\left(n+d_2/d\right)^3}\right]. \tag{S8}$$

The Hamiltonian (S7) concludes the dipolar excitations in a dimerized chain with long-range interactions. In the nearest-neighbor coupling approximation, the intrasublattice Hamiltonian (S5) is completely neglected, while the intersublattice Hamiltonian (S6) is restricted to $m=n$ and $m=n+1$, respectively. Therefore, one can get

$$f_{k_x}=0\quad\text{and}\quad g_{k_x}=\frac{1}{\left(d_1/d\right)^3}+\frac{\mathrm{e}^{-\mathrm{i}k_xd}}{\left(d_2/d\right)^3}. \tag{S9}$$

To emulate the Su-Schrieffer–Heeger (SSH) model, we define $g_{k_x}=t_1/\Omega+(t_2/\Omega)\,e^{-ik_xd}$, where $t_1=\Omega\,(d/d_1)^3$ [$t_2=\Omega\,(d/d_2)^3$] represents intracell coupling [intercell coupling]. So the Hamiltonian (S7) can be rewritten as

$$H_{\mathrm{dp}}=\sum_{k_x}\Big\{\omega_0\left(a_{k_x}^\dagger a_{k_x}+b_{k_x}^\dagger b_{k_x}\right)+\left(t_1+t_2\mathrm{e}^{-\mathrm{i}k_xd}\right)\left[a_{k_x}^\dagger\left(b_{k_x}+b_{-k_x}^\dagger\right)\right]+\left(t_1+t_2\mathrm{e}^{\mathrm{i}k_xd}\right)\left[\left(b_{k_x}^\dagger+b_{-k_x}\right)a_{k_x}\right]\Big\}, \tag{S10}$$

In summary, it can be concluded that

$$H_{\mathrm{dp}}=\begin{pmatrix}\omega_0 & t_1+t_2e^{-ik_xd}\\ t_1+t_2e^{ik_xd} & \omega_0\end{pmatrix}, \tag{S11}$$

see the Eq. (1) in the main text.

1.2 The photonic Hamiltonian

In Eq. (S1), the photonic Hamiltonian is read as

$$H_{\mathrm{ph}}=\sum_{\mathbf{q},\boldsymbol{\lambda}_\mathbf{q}}\omega_\mathbf{q}^{\mathrm{ph}}c_{\mathbf{q},\boldsymbol{\lambda}_\mathbf{q}}^\dagger c_{\mathbf{q},\boldsymbol{\lambda}_\mathbf{q}}, \tag{S12}$$

where $c_{\mathbf{q},\boldsymbol{\lambda}_\mathbf{q}}\left(c_{\mathbf{q},\boldsymbol{\lambda}_\mathbf{q}}^\dagger\right)$ annihilates (creates) a photon with wavevector $\mathbf{q}$ and two possible transverse polarizations labeled as $\boldsymbol{\lambda}_\mathbf{q}=\{\mathbf{1}_\mathbf{q},\mathbf{2}_\mathbf{q}\}$. The photonic mode frequency is $\omega_\mathbf{q}^{\mathrm{ph}}=c|\mathbf{q}|$, where c is the speed of light in vacuum and the associated vector potential

$$\mathbf{A}(\mathbf{r})=\sum_{\mathbf{q},\boldsymbol{\lambda}_\mathbf{q}}\sqrt{\frac{2\pi c^2}{\omega^{\mathrm{ph}}_\mathbf{q}}}\left(\mathbf{f}_{\mathbf{q},\boldsymbol{\lambda}_\mathbf{q}}(\mathbf{r})c_{\mathbf{q},\boldsymbol{\lambda}_\mathbf{q}}+\mathbf{f}^*_{\mathbf{q},\boldsymbol{\lambda}_\mathbf{q}}(\mathbf{r})c^\dagger_{\mathbf{q},\boldsymbol{\lambda}_\mathbf{q}}\right), \tag{S13}$$

where $\mathbf{f}_{\mathbf{q},\boldsymbol{\lambda}_\mathbf{q}}(\mathbf{r})$ are the mode functions of the photonic cavity [2]. In a cuboid cavity of dimensions $L_x \times L_y \times L_z$, see Supplementary Fig. 1, the photon wavevector in spherical coordinates read as

$$\mathbf{q}=\begin{pmatrix} q_x \\ q_y \\ q_z \end{pmatrix}=\begin{pmatrix} q_x \\ \frac{\pi n_y}{L_y} \\ \frac{\pi n_z}{L_z} \end{pmatrix}=q\begin{pmatrix} \sin\theta_\mathbf{q}\cos\phi_\mathbf{q} \\ \sin\theta_\mathbf{q}\sin\phi_\mathbf{q} \\ \cos\theta_\mathbf{q} \end{pmatrix}, \tag{S14}$$

where the component q_x is continuous since we take the length L_x of the cavity to be tending towards infinity, n_y and n_z are non-negative integers, arising from hard-wall boundary conditions of the cavity. The spatial profiles of the mode functions then read as [3]

$$\mathbf{f}_{\mathbf{q},\boldsymbol{\lambda}_\mathbf{q}}(\mathbf{r})=\sqrt{\frac{2}{V}}\begin{pmatrix} \sin(q_y y)\sin(q_z z) & (\boldsymbol{\lambda}_\mathbf{q}\cdot\mathbf{x}) \\ \cos(q_y y)\sin(q_z z) & (\boldsymbol{\lambda}_\mathbf{q}\cdot\mathbf{y}) \\ \sin(q_y y)\cos(q_z z) & (\boldsymbol{\lambda}_\mathbf{q}\cdot\mathbf{z}) \end{pmatrix}\mathrm{e}^{\mathrm{i}q_x x}, \tag{S15}$$

with the cavity volume $V=L_xL_yL_z$. The two-photon polarizations $\boldsymbol{\lambda}_\mathbf{q}=\{\mathbf{1}_\mathbf{q},\mathbf{2}_\mathbf{q}\}$ are given by

$$\mathbf{1}_\mathbf{q}=\begin{pmatrix} \cos\theta_\mathbf{q}\cos\phi_\mathbf{q} \\ \cos\theta_\mathbf{q}\sin\phi_\mathbf{q} \\ -\sin\theta_\mathbf{q} \end{pmatrix} \quad \text{and} \quad \mathbf{2}_\mathbf{q}=\begin{pmatrix} -\sin\phi_\mathbf{q} \\ \cos\phi_\mathbf{q} \\ 0 \end{pmatrix}. \tag{S16}$$

The photonic mode frequency in Eq. (S12) reads as

$$\omega^{\mathrm{ph}}_\mathbf{q}=\omega^{\mathrm{ph}}_{q_x,n_y,n_z}=c\sqrt{q_x^2+\left(\frac{\pi n_y}{L_y}\right)^2+\left(\frac{\pi n_z}{L_z}\right)^2}. \tag{S17}$$

In our model, we have $L_x \gg L_y \gg L_z$, and the mode function $\mathbf{f}_{\mathbf{q}\mathbf{2}_\mathbf{q}}(\mathbf{r})$ in Eq. (S15) can be simplified as

$$\mathbf{f}_{q_x,n_y}(\mathbf{r})=\sqrt{\frac{2}{V}}\begin{pmatrix} 0 \\ 0 \\ \sin\left(\frac{\pi n_y y}{L_y}\right) \end{pmatrix}\mathrm{e}^{\mathrm{i}q_x x}. \tag{S18}$$

1.3 The light-matter interaction Hamiltonian

The light-matter interaction Hamiltonian in Eq. (S1) involves a paramagnetic term $H_{\Pi\cdot\mathbf{A}}$ and a diamagnetic term $H_{\mathbf{A}^2}$, such that [4]

$$H_{\mathrm{dp-ph}}=H_{\mathbf{\Pi}\cdot\mathbf{A}}+H_{\mathbf{A}^2}. \tag{S19}$$

In the long-wavelength approximation $|\mathbf{q}|a \ll 1$, we have explicitly

$$H_{\mathbf{\Pi}\cdot\mathbf{A}}=\sum_{n=1}^{N}\sum_{s=A,B}\frac{Q}{Mc_0}\mathbf{\Pi}_{n,s}\cdot\mathbf{A}\left(\mathbf{r}_{n,s}\right). \tag{S20}$$

and

$$H_{\mathbf{A}^2}=\sum_{n=1}^{N}\sum_{s=A,B}\frac{Q^2}{2Mc_0^2}\mathbf{A}^2\left(\mathbf{r}_{n,s}\right). \tag{S21}$$

The dipole momenta on the A and B sublattices in the dimer n are

$$\mathbf{\Pi}_{n,A}=\mathrm{i}\sqrt{\frac{M\omega_0}{2}}\left(a_n^\dagger-a_n\right)\mathbf{z} \quad \text{and} \quad \mathbf{\Pi}_{n,B}=\mathrm{i}\sqrt{\frac{M\omega_0}{2}}\left(b_n^\dagger-b_n\right)\mathbf{z}, \tag{S22}$$

and the positions of the dips are denoted as

$$\mathbf{r}_{n,A} = \begin{pmatrix} nd - \frac{d_1}{2} \\ \frac{L_y}{2} \\ \frac{L_z}{2} \end{pmatrix} \quad \text{and} \quad \mathbf{r}_{n,B} = \begin{pmatrix} nd + \frac{d_1}{2} \\ \frac{L_y}{2} \\ \frac{L_z}{2} \end{pmatrix}. \tag{S23}$$

In the single photonic band regime, the vector potential of Eq. (S13) becomes

$$\mathbf{A}(x) = \sum_{q_x} \sqrt{\frac{4\pi c^2}{V\omega_{q_x}^{\mathrm{ph}}}} \left(c_{q_x} \mathrm{e}^{\mathrm{i}q_x x} + c_{q_x}^{\dagger} \mathrm{e}^{-\mathrm{i}q_x x}\right) \mathbf{z}. \tag{S24}$$

By substituting the momenta (S22) and the vector potential (S24), the paramagnetic Hamiltonian (S20) at the positions (S23) reads as

$$H_{\mathbf{\Pi}\cdot\mathbf{A}} = \sum_{k_x} \mathrm{i}\xi_{k_x} \left[\left(a_{k_x}^{\dagger} \mathrm{e}^{-\mathrm{i}k_x d_1/2} + b_{k_x}^{\dagger} \mathrm{e}^{\mathrm{i}k_x d_1/2}\right)\left(c_{k_x} + c_{-k_x}^{\dagger}\right) - \left(c_{k_x}^{\dagger} + c_{-k_x}\right)\left(a_{k_x} \mathrm{e}^{\mathrm{i}k_x d_1/2} + b_{k_x} \mathrm{e}^{-\mathrm{i}k_x d_1/2}\right)\right], \tag{S25}$$

and the diamagnetic Hamiltonian (S21) reads as

$$H_{\mathbf{A}^2} = \sum_{k_x} \frac{2\xi_{k_x}^2}{\omega_0} \left[c_{k_x}^{\dagger}\left(c_{k_x} + c_{-k_x}^{\dagger}\right) + \left(c_{k_x}^{\dagger} + c_{-k_x}\right) c_{k_x}\right], \tag{S26}$$

with the light-matter coupling constant

$$\xi_{k_x} = \omega_0 \left(\frac{2\pi a^3}{L_y L_z d} \frac{\omega_0}{\omega_{k_x}^{\mathrm{ph}}}\right)^{1/2}. \tag{S27}$$

where $a = \left(\frac{Q^2}{M\omega_0^2}\right)^{1/3}$ is the reparameterized length scale and $\omega_{k_x}^{\mathrm{ph}} = c\sqrt{{k_x}^2 + \left(\frac{\pi}{L_y}\right)^2}$ is the cavity photon frequency. We depict the amplitude of light-matter coupling as a function of cavity width L_y in Supplementary Fig. 2, as derived from Eq. (S27). It can be seen that the amplitude of light-matter interaction decreases with increasing cavity width L_y, except for the point $k_x = 0$.

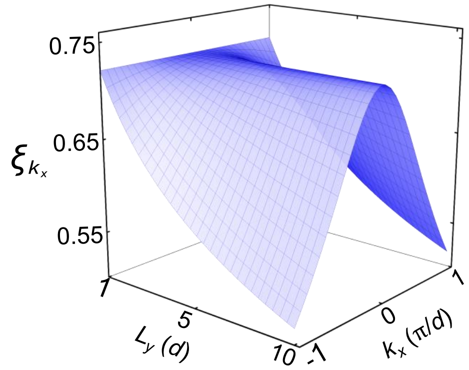

Supplementary Fig. 2 Amplitude of light-matter coupling as a function of cavity width L_y.

Generally, the photonic band for a long cavity with periodic boundary condition along the x direction can be expressed as $\omega_{k_x,n_y,n_z}^{ph} = c_0\sqrt{{k_x}^2 + \left(\frac{n_y\pi}{L_y}\right)^2 + \left(\frac{n_z\pi}{L_z}\right)^2}$, where n_y and n_z are the nth order mode along the y and z directions, respectively. The long cavity in our work satisfies $L_x \gg L_y \gg L_z$, implying that the fundamental photonic cavity mode along the z directions i.e., $(n_y, n_y) = (0, 1)$ for $\omega_{k_x,0,1}^{ph}$, possess significantly higher frequencies than the fundamental photonic band along the y direction, i.e., $(n_y, n_z) = (1, 0)$ for $\omega_{k_x,0,1}^{ph}$.Since we focus on the light-matter interaction between the fundamental modes of the MHRs and the fundamental cavity modes. thus we choose the fundamental cavity band with $(n_y, n_z) = (1, 0)$, which possesses closest frequency regimewhich possesses closest frequency regime $\omega_{k_x,1,0}^{ph} = c_0\sqrt{{k_x}^2 + \left(\frac{\pi}{L_y}\right)^2}$, with the fundamental bands of the SSH chain of MHRs. Therefore, in this case, the k_z can be neglected.

1.4 The polaritonic Hamiltonian

With total photonic operators being considered, the polaritonic Hamiltonian can be written as

$$H_{\mathrm{pol}} = \sum_{k_x} \boldsymbol{\psi}_{k_x}^{\mathrm{pol},\dagger} \mathcal{H}_{k_x}^{\mathrm{pol}} \boldsymbol{\psi}_{k_x}^{\mathrm{pol}}, \tag{S28}$$

in the basis $\boldsymbol{\psi}_{k_x}^{\mathrm{pol}} = \left(a_{k_x}, b_{k_x}, c_{k_x}^{(0)}, c_{k_x}^{(1)}, c_{k_x}^{(-1)}, \cdots\right)$ and the polaritonic Bloch Hamiltonian reads as

$$H_{k_x}^{\mathrm{pol}} = \begin{pmatrix} \omega_0 & \Omega g_{k_x} & \mathrm{i}\xi_{k_x}^{(0)}\mathrm{e}^{-\mathrm{i}\chi_{k_x}^{(0)}} & \mathrm{i}\xi_{k_x}^{(1)}\mathrm{e}^{-\mathrm{i}\chi_{k_x}^{(1)}} & \mathrm{i}\xi_{k_x}^{(-1)}\mathrm{e}^{-\mathrm{i}\chi_{k_x}^{(-1)}} & \cdots \\ \Omega g_{k_x}^{*} & \omega_0 & \mathrm{i}\xi_{k_x}^{(0)}\mathrm{e}^{\mathrm{i}\chi_{k_x}^{(0)}} & \mathrm{i}\xi_{k_x}^{(1)}\mathrm{e}^{\mathrm{i}\chi_{k_x}^{(1)}} & \mathrm{i}\xi_{k_x}^{(-1)}\mathrm{e}^{\mathrm{i}\chi_{k_x}^{(-1)}} & \cdots \\ -\mathrm{i}\xi_{k_x}^{(0)}\mathrm{e}^{\mathrm{i}\chi_{k_x}^{(0)}} & -\mathrm{i}\xi_{k_x}^{(0)}\mathrm{e}^{-\mathrm{i}\chi_{k_x}^{(0)}} & \omega_{k_x}^{\mathrm{ph}(0)} & 0 & 0 & \cdots \\ -\mathrm{i}\xi_{k_x}^{(1)}\mathrm{e}^{\mathrm{i}\chi_{k_x}^{(1)}} & -\mathrm{i}\xi_{k_x}^{(1)}\mathrm{e}^{-\mathrm{i}\chi_{k_x}^{(1)}} & 0 & \omega_{k_x}^{\mathrm{ph}(1)} & 0 & \\ -\mathrm{i}\xi_{k_x}^{(-1)}\mathrm{e}^{\mathrm{i}\chi_{k_x}^{(-1)}} & -\mathrm{i}\xi_{k_x}^{(-1)}\mathrm{e}^{-\mathrm{i}\chi_{k_x}^{(-1)}} & 0 & 0 & \omega_{k_x}^{\mathrm{ph}(-1)} & \\ \vdots & \vdots & \vdots & & & \ddots \end{pmatrix}, \tag{S29}$$

with the general photon dispersion

$$\omega_{k_x}^{\mathrm{ph}(m)} = c\sqrt{\left(k_x - \frac{2\pi m}{d}\right)^2 + \left(\frac{\pi}{L_y}\right)^2}, \quad m = 0, \pm 1, \pm 2, \ldots, \tag{S30}$$

the light-matter coupling constant

$$\xi_{k_x}^{(m)} = \omega_0 \left(\frac{2\pi a^3}{L_x L_y d} \frac{\omega_0}{\omega_{k_x}^{\mathrm{ph}(m)}}\right)^{1/2}, \tag{S31}$$

and χ_{k_x}

$$\chi_{k_x}^{(m)} = \frac{d_1}{d}\left(\frac{k_x d}{2} - m\pi\right). \tag{S32}$$

The quantities in the winding vector read

$$\omega_{0,j} = \omega_0 + \sum_{m=-\infty}^{+\infty} \frac{\xi_{k_x}^{(m)^2}}{\omega_{k_x,j}^{\mathrm{pol}} - \omega_{k_x}^{\mathrm{ph}(m)}}, \quad h_{k_x,j} = g_{k_x} + \frac{1}{\Omega}\sum_{m=-\infty}^{+\infty} \frac{\mathrm{e}^{-2\mathrm{i}\chi_{k_x}^{(m)}}\xi_{k_x}^{(m)^2}}{\omega_{k_x,j}^{\mathrm{pol}} - \omega_{k_x}^{\mathrm{ph}(m)}}, \tag{S33}$$

with the polaritonic eigenfrequencies $\omega_{k_x,j}^{\mathrm{pol}}$.

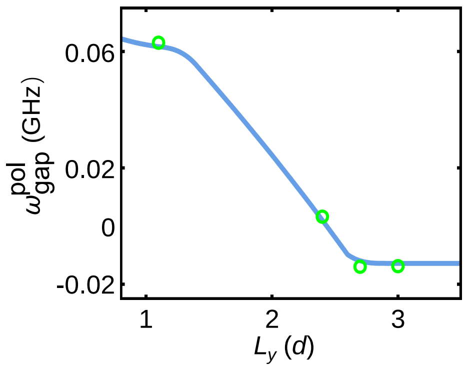

Supplementary Fig. 3 Dipole-photon energy detuning $\omega_{\mathrm{gap}}^{\mathrm{pol}}$ as a function of cavity width L_y.

1.5 Dipole-photon energy detuning

The variation of dipole-photon energy detuning $\omega_{\text{gap}}^{\text{pol}} = \min\{\omega_{\text{U}}^{\text{pol}}\} - \max\{\omega_{\text{P}}^{\text{pol}}\}$ [1] at either $k_x = \pi$ or 0 as a function of cavity width L_y is plotted in Supplementary Fig. 3 with the blue line (green circles) represents the simulation (measured) results, respectively. It can be seen that as the cavity width increases, the dipole-photon energy detuning $\omega_{gap}^{\text{pol}}$ gradually decreases.

Supplementary Note 2. Phase transitions

In this section, we analyze the phase transition of the models in the rotating-wave approximation (RWA). The RWA is appropriate when the frequencies of all modes are close to resonance.

2.1 SU(3) polaritonic model

After disregarding the counter-rotating terms which correspond to the approximation for $\Omega \ll \omega_0$, the polaritonic Hamiltonian denotes as

$$\begin{aligned} H_{\text{pol}}^{\text{RWA}} = \sum_{k_x} \Big[& \omega_0 \left(a_{k_x}^\dagger a_{k_x} + b_{k_x}^\dagger b_{k_x} \right) + \Omega \left(g_{k_x} a_{k_x}^\dagger b_{k_x} + g_{k_x}^* b_{k_x}^\dagger a_{k_x} \right) + \omega_{k_x}^{\text{ph}} c_{k_x}^\dagger c_{k_x} \\ & + \text{i}\xi_{k_x} \left(a_{k_x}^\dagger \text{e}^{-\text{i}k_x d_1/2} + b_{k_x}^\dagger \text{e}^{\text{i}k_x d_1/2} \right) c_{k_x} - \text{i}\xi_{k_x} c_{k_x}^\dagger \left(a_{k_x} \text{e}^{\text{i}k_x d_1/2} + b_{k_x} \text{e}^{-\text{i}k_x d_1/2} \right) \Big], \end{aligned} \tag{S34}$$

and can be alternatively written as

$$H_{\text{pol}}^{\text{RWA}} = \sum_{k_x} \boldsymbol{\psi}_{k_x}^{\text{pol},\dagger} \mathcal{H}_{k_x}^{\text{pol,RWA}} \boldsymbol{\psi}_{k_x}^{\text{pol}}, \tag{S35}$$

in the basis $\boldsymbol{\psi}_{k_x}^{\text{pol}} = (a_{k_x}, b_{k_x}, c_{k_x})$ with the polaritonic Bloch Hamiltonian

$$H_{k_x}^{\text{pol,RWA}} = \begin{pmatrix} \omega_0 & \Omega g_{k_x} & \text{i}\xi_{k_x} \text{e}^{-\text{i}\chi_{k_x}} \\ \Omega g_{k_x}^* & \omega_0 & \text{i}\xi_{k_x} \text{e}^{\text{i}\chi_{k_x}} \\ -\text{i}\xi_{k_x} \text{e}^{\text{i}\chi_{k_x}} & -\text{i}\xi_{k_x} \text{e}^{-\text{i}\chi_{k_x}} & \omega_{k_x}^{\text{ph}} \end{pmatrix}. \tag{S36}$$

Equation (S36) can be rewritten as

$$H_{k_x}^{\text{pol,RWA}} = \begin{pmatrix} \omega_0 & t_1 + t_2 e^{-ik_x d} & \text{i}\xi_{k_x} e^{-\text{i}\chi_{k_x}} \\ t_1 + t_2 e^{ik_x d} & \omega_0 & \text{i}\xi_{k_x} e^{\text{i}\chi_{k_x}} \\ -\text{i}\xi_{k_x} e^{\text{i}\chi_{k_x}} & -\text{i}\xi_{k_x} e^{-\text{i}\chi_{k_x}} & \omega_{k_x}^{\text{ph}} \end{pmatrix}, \tag{S37}$$

corresponding to Eq. (2) in the main text (which is $\mathcal{H}_{\text{pol}}$ as the subscripts k_x are dropped for simplicity). The polaritonic Zak phase gives

$$\theta_{\text{pol}}^{\text{Zak}} = \text{i} \int_{-\pi/d}^{+\pi/d} dk_x \left\langle \psi_{k_x,j}^{\text{pol}} \left| \partial_{k_x} \right| \psi_{k_x,j}^{\text{pol}} \right\rangle, \tag{S38}$$

with the polariton band index $j = \{1, 2, 3\}$.

2.2 SU(2) polaritonic model

In Sec. 2. 2.1, we have shown how the polaritonic Bloch Hamiltonian (S36) can be written as a 3×3 matrix in the SU(3) group. By eliminating the photonic degree of freedom, we rewrite the Hamiltonian (S36) as an effective 2×2 matrix Hamiltonian in the SU(2) group:

$$H_{k_x,j}^{\text{pol,RWA}} = \begin{pmatrix} \omega_{0,j} & \Omega h_{k_x,j} \\ \Omega h_{k_x,j}^* & \omega_{0,j} \end{pmatrix} = \omega_{0,j} \mathcal{I}_0 + \boldsymbol{\sigma} \cdot \boldsymbol{w}_{k_x,j}, \tag{S39}$$

and where the renormalized on-diagonal frequency $\omega_{0,j}$ and off-diagonal quantity $h_{k_x,j}$ are defined via

$$\omega_{0,j} = \omega_0 + \frac{\xi_{k_x}^2}{\omega_{k_x,j}^{\mathrm{pol}} - \omega_{k_x}^{\mathrm{ph}}}, \quad h_{k_x,j} = g_{k_x} + \frac{\mathrm{e}^{-2\mathrm{i}\chi_{k_x}}}{\Omega} \frac{\xi_{k_x}^2}{\omega_{k_x,j}^{\mathrm{pol}} - \omega_{k_x}^{\mathrm{ph}}}, \tag{S40}$$

with the three polaritonic eigenfrequencies $\omega_{k_x,j}^{\mathrm{pol}}$ [by taking the dominant $m = 0$ in Eq. (S32)]. Now we have an effective winding vector $\boldsymbol{w}_{k_x,j}$, with the components

$$w_{k_x,jx} = \Omega\,|h_{k_x,j}|\cos\varphi_{k_x,j}, \quad w_{k_x,jy} = -\Omega\,|h_{k_x,j}|\sin\varphi_{k_x,j}, \quad \mathrm{e}^{\mathrm{i}\varphi_{k_x,j}} = \frac{h_{k_x,j}}{|h_{k_x,j}|}. \tag{S41}$$

Supplementary Note 3. Simulation and Experimental details

In this section, we introduce the experimental details of our scheme, including the design of the microwave helical resonator, the definition of the cavity mode, and the measurement of the edge states and the Zak phase.

3.1 The microwave helical resonator mode

The MHRs exhibit induced dipole moments that are oriented along the direction of their helical axis, thereby introducing dipole interactions [1]. To demonstrate the field distributions of the coupled modes of two microwave helical resonators, Supplementary Fig. 4(a)-4(b) presents the front views of the E_z field distributions of the symmetric [Supplementary Fig. 4(a)] and antisymmetric [Supplementary Fig. 4(b)] coupled modes [referring to the points B and C in Fig. 2(f) of the main text], revealing the dipolar characteristic of the coupled modes.

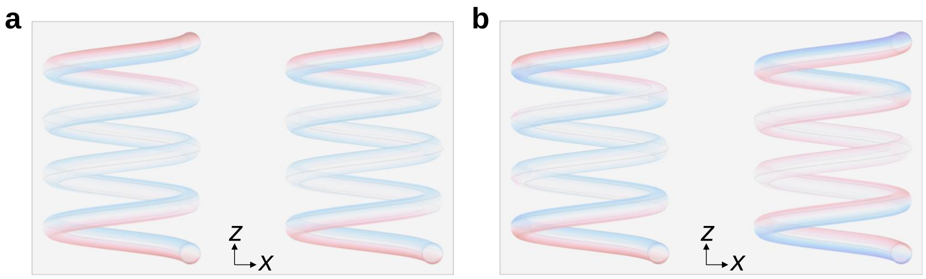

Supplementary Fig. 4 The E_z field distribution of the symmetric (a) and antisymmetric (b) couple dipolar modes of two microwave helical resonators.

3.2 The cavity mode

We solve the cavity modes using both the numerical simulations (COMSOL Multiphysics) and theoretical calculation with the analytical equation of cavity modes $\omega^{ph}_{k_x,n_y,0} = c_0\sqrt{{k_x}^2 + \left(\frac{n_y\pi}{L_y}\right)^2}$. Supplementary Fig. 5a-5d presents the simulated (solid lines) and analytical (triangle symbols) band structures of the cavity modes for different cavity widths, respectively. Each cavity can support multiple modes simultaneously ($n_y = 1, 2, 3\ldots$), here we only focus on the fundamental mode (highlighted in green), which couples with the fundamental modes of an SSH chain of MHRs highlighted by red and orange lines in Supplementary Fig. 5e.

The resonance frequency (f_r) of TE(1,1,0) mode the cavity can be determined using Eq (S42) [5]

$$(f_r)^{\mathrm{TE}}_{1,1,0} = 0.5c_0\sqrt{(\frac{1}{d})^2 + (\frac{1}{L_y})^2}. \tag{S42}$$

The quality factor(Q_{fac}) of the cavity can be determined using Eq (S43) [5]

$$Q_{fac} = \frac{\pi\eta}{2R_S}[\frac{L_z(L_y^2+d^2)^{3/2}}{L_yd(L_y^2+d^2)+2L_z(L_y^3+d^3)}]. \tag{S43}$$

where d is lattice constant, L_y, and L_z, are the width, height, respectively. $\eta = 377$ is the intrinsic impedance of the medium. c_0 is the speed of light in the vacuum. $R_S = 0.0254$ is the surface resistance. For the cavity with a width of $L_y = 1.1d$, the calculated resonance frequency is $f_r = 5.065$ GHz and the quality factor is Q_{fac}=8790.53 [5].

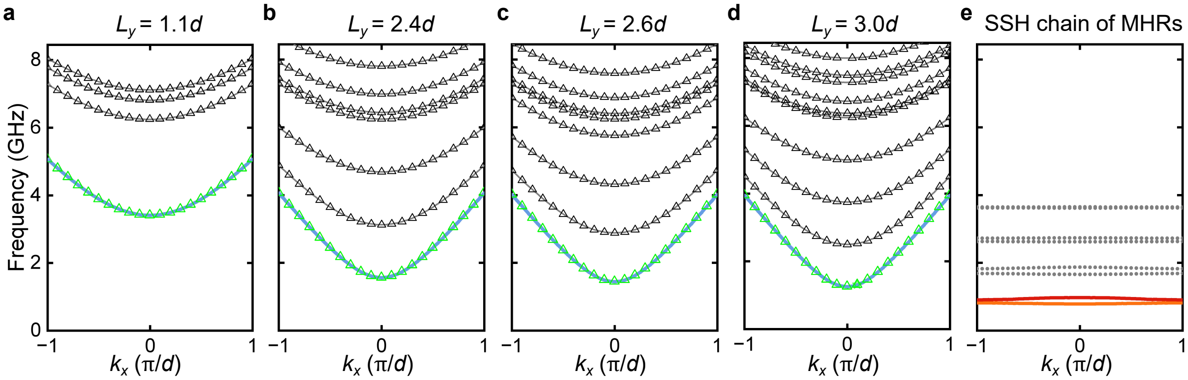

Supplementary Fig. 5 The mode of cavity and SSH chain of MHRs. a-d Simulated (solid lines) and analytical (triangle symbols) cavity modes for different cavity widths $L_y = 1.1d$ **a**, $2.4d$ **b**, $2.6d$ **c**, and $3d$ **d**, respectively. The fundamental modes are highlighted in green color. **e** Simulated band structures of an SSH chain of MHRs whose fundamental modes are highlighted in red and orange lines.

3.3 The experimental scheme

In the microwave experiment, we use a vector network analyzer (VNA) to generate and detect the microwave signals. As shown in Supplementary Fig. 6, two dipole antennas are adopted as the source to excite the bulk and edge states and the probe to measure E_z field distributions (both amplitude and phase) of the experimental sample. By performing Fast Fourier Transformation (FFT) on the measured E_z field distributions, we can obtain the measured dispersion. Moreover, the resolution of the measured E_z field distributions in real space is 4.5×5 mm (i.e., 10×7 points within each unit cell), and there are 15 points in the momentum-space band structure corresponding to the 15 unit cells of the sample.

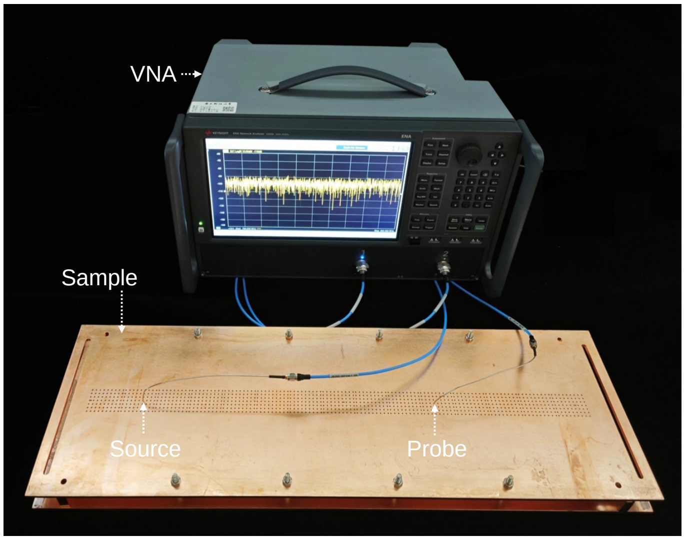

Supplementary Fig. 6 A photograph of experimental setup.

3.4 The simulated and measured amplitude distributions of the right topological edge states

Supplementary Fig. 7 shows the simulated (Supplementary Fig. 7a) and measured (Supplementary Fig. 7b) amplitude distributions of $|E_z|$ field of the right topological edge states as a function of the cavity length L_x, which exhibit similar behavior with those of the left topological edge states in the main manuscript.

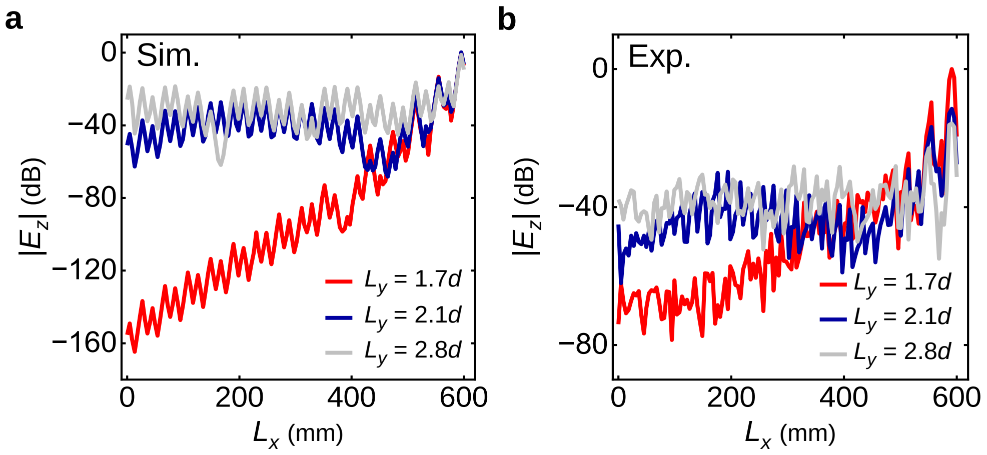

Supplementary Fig. 7 Simulated (a) and measured (b) amplitude distributions of $|E_z|$ field of the right topological edge states as a function of the cavity length L_x with different cavity widths L_y =1.7d (red line), 2.1d (blue line), and 2.8d (gray line), respectively.

[1] C. A. Downing, T. J. Sturges, G. Weick, M. Stobińska, and L. Martín-Moreno, Topological phases of polaritons in a cavity waveguide, Phys. Rev. Lett. **123**, 217401 (2019).

[2] P. W. Milonni, *The quantum vacuum: an introduction to quantum electrodynamics* (Academic press, 2013).

[3] K. Kakazu and Y. S. Kim, Quantization of electromagnetic fields in cavities and spontaneous emission, Phys. Rev. A **50**, 1830 (1994).

[4] C.-R. Mann, T. J. Sturges, G. Weick, W. L. Barnes, and E. Mariani, Manipulating type-I and type-II Dirac polaritons in cavity-embedded honeycomb metasurfaces, Nat. Commun. **9**, 2194 (2018).

[5] C. A. Balanis, *Advanced engineering electromagnetics*, 2nd ed. (John Wiley & Sons, Hoboken, N.J, 2012).
